# Synthetic Gene Circuits Combining CRISPR Interference and CRISPR Activation in *E. coli*: Importance of Equal Guide RNA Binding Affinities to Avoid Context-Dependent Effects

**DOI:** 10.1021/acssynbio.3c00375

**Published:** 2023-10-09

**Authors:** Içvara Barbier, Hadiastri Kusumawardhani, Lakshya Chauhan, Pradyumna Vinod Harlapur, Mohit Kumar Jolly, Yolanda Schaerli

**Affiliations:** † Department of Fundamental Microbiology, University of Lausanne, 1015 Lausanne, Switzerland; ‡ Department of Bioengineering, Indian Institute of Science, 560012 Bengaluru, India

**Keywords:** bacterial synthetic biology, synthetic gene circuits, CRISPR interference, CRISPR activation, resource competition, dCas9

## Abstract

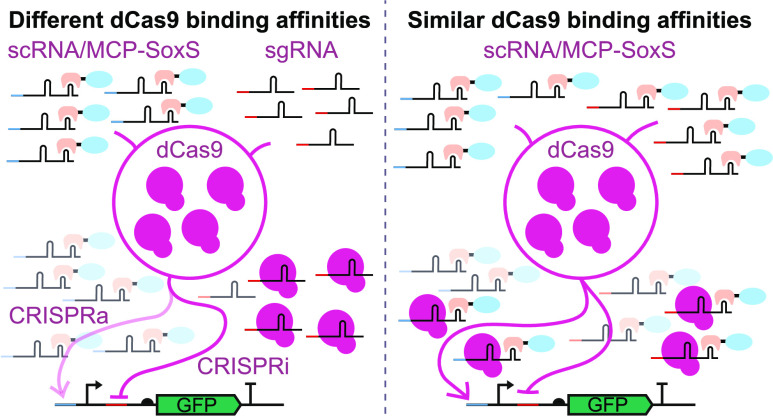

Gene expression control based on clustered regularly interspaced short palindromic repeats (CRISPR) has emerged as a powerful approach for constructing synthetic gene circuits. While the use of CRISPR interference (CRISPRi) is already well-established in prokaryotic circuits, CRISPR activation (CRISPRa) is less mature, and a combination of the two in the same circuits is only just emerging. Here, we report that combining CRISPRi with SoxS-based CRISPRa in *Escherichia coli* can lead to context-dependent effects due to different affinities in the formation of CRISPRa and CRISPRi complexes, resulting in loss of predictable behavior. We show that this effect can be avoided by using the same scaffold guide RNA structure for both complexes.

## Introduction

Synthetic biologists are building synthetic gene regulatory networks (GRNs) to decipher nature’s design principles^1,2^ and to provide new solutions to biomedical,^3^ agricultural,^4^ industrial,^5^ and environmental challenges.^6^ Despite impressive progress in constructing synthetic circuits,^7,8^ the complexity of gene regulatory networks that has been achieved is still rather limited.^9−11^ Challenges to overcome include metabolic burden, resource competition, a limited number of well-categorized parts, cross-talk between parts, and context-dependent effects, leading to low modularity and scalability. While most synthetic circuits built so far have made use of protein transcription factors to regulate gene expression, clustered regularly interspaced short palindromic repeats (CRISPR)-based genetic regulation has the potential to address many of the current limitations.^12^ The advantages of CRISPR-based gene regulation tools compared to circuits based on protein transcription factors include decreased cross-talk between parts due to highly specific RNA–DNA interactions, reduced metabolic burden coming from protein production, and straightforward design of a virtually unlimited number of orthogonal versions.^12^

The strategies for transcriptional regulation based on CRISPR are known as CRISPR interference (CRISPRi)^13^ and CRISPR activation (CRISPRa).^14,15^ In bacteria, the repression system uses a single guide RNA (sgRNA), which is composed of a target-specific sequence and a sequence that recruits a catalytically inactive version of Cas9 (dCas9). The complex is targeted to a promoter or a coding sequence to inhibit transcription. In contrast, for CRISPRa, dCas9 is targeted upstream of the promoter, and it requires, in addition, an activator protein to recruit the RNA polymerase.^14,15^ In prokaryotes, the activator protein can be directly fused to dCas9,^16−21^ or alternatively, the sgRNA can be extended with a protein-recruiting RNA scaffold that recruits the transcriptional activator.^22−26^ Probably the best characterized bacterial CRISPRa system is based on a so-called scaffold RNA (scRNA), where the sgRNA is modified to include a 3′ MS2 hairpin. This hairpin recruits the MS2 coat protein (MCP) that is fused to the transcriptional activator SoxS.^22−25^

We recently showed that CRISPRi can be used to build dynamic and multistable synthetic circuits.^27^ Extending such circuits with CRISPRa has the potential to further increase the complexity of synthetic gene regulation programs. Here, we show that a combination of CRISPRi with CRISPRa based on scRNA and SoxS can lead to strong context-dependent effects. Specifically, the strength of activation mediated by scRNA was strongly influenced by the concurrent expression of an sgRNA. We hypothesized that this phenomenon was caused by sgRNA and scRNA competing for the limited pool of dCas9 and by their differential affinities to dCas9. This hypothesis was supported by a mathematical model. The model also suggested ways to circumvent this problem. We then experimentally reduced this context-dependent effect by using scRNAs for both repression and activation, thus improving the predictability of synthetic CRISPRa/i circuits.

## Results

### Implementing CRISPRa

We implemented CRISPRa using our previously developed plasmid architecture and cloning strategy,^28^ which we had employed to construct multistable and dynamic CRISPRi-based circuits.^27^ Our CRISPRi system is composed of two plasmids. The first plasmid (colA ori) harbors the designed circuit with up to three nodes, one of which is arabinose-inducible via a pBAD promoter. From the second plasmid (CDF ori), we constitutively express dCas9 and Csy4. We use Csy4 RNase-processing to release parts that are transcribed together in the same operon to act independently once transcribed, such as sgRNAs/scRNAs for CRISPRi/a and mRNAs encoding a fluorescent reporter.

**Figure 1:**
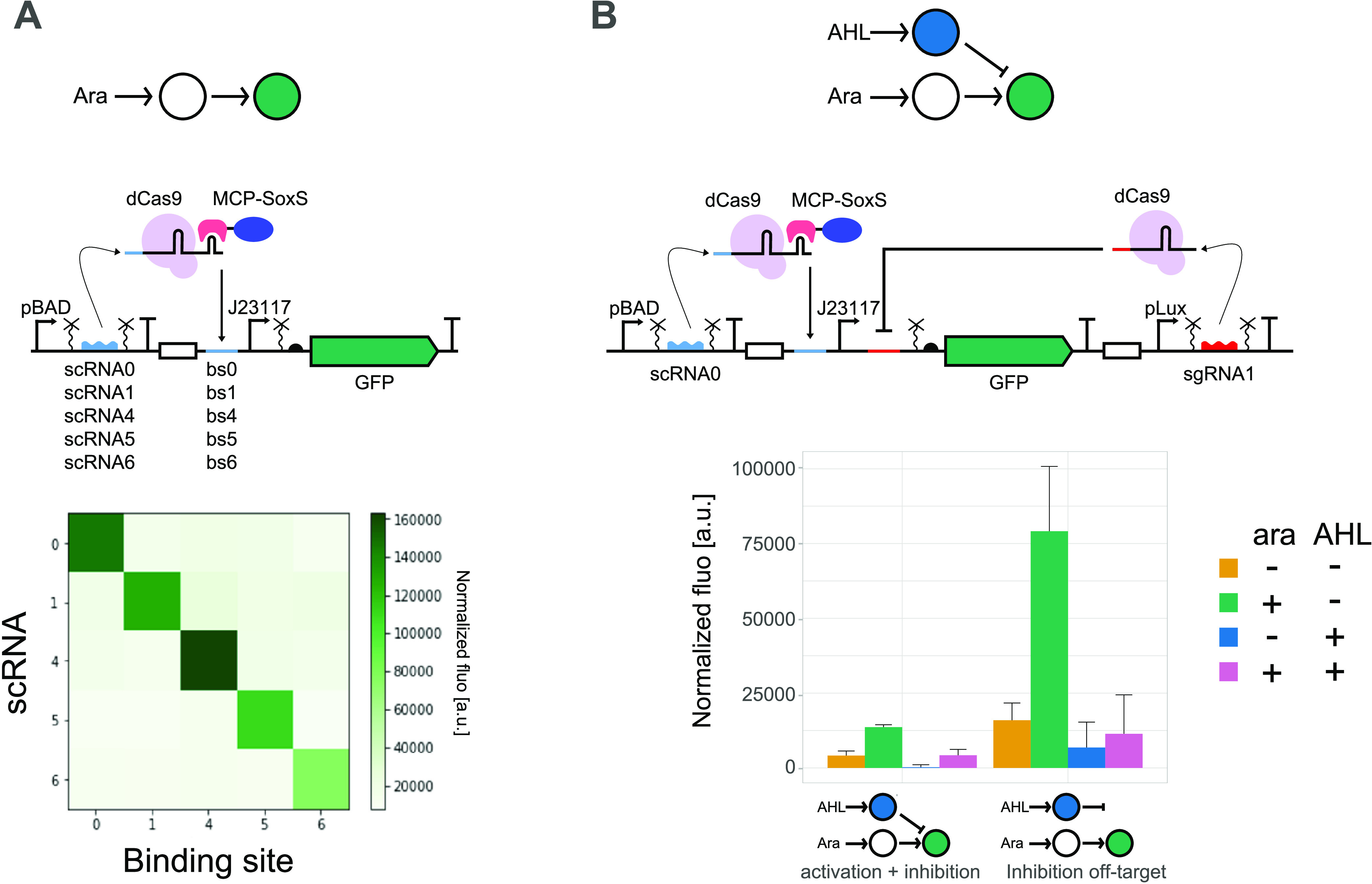
CRISPRa orthogonality and combination with CRISPRi. (A) CRISPRa orthogonality. Top: schematic representation of the circuit and details of the circuit design. Symbols according to SBOL standard.^29^ Bottom: orthogonality heatmap of GFP fluorescence normalized by the absorbance at 0.2% arabinose with different combinations of scRNAs and binding sites, as represented in the figure. (B) Combination of CRISPRa and CRISPRi. Top: details of the circuit design. Middle: schematic representation of the circuit. Bottom: bar plots represent the GFP fluorescence normalized by the absorbance in the absence or presence of arabinose (0 and 0.2%) and AHL (0 and 0.1 μM). These data show very low target activation and incorrect off-target control behavior, where sgRNA induction with AHL should not affect GFP production. Binding site number 2 was used for off-target inhibition. Mean and standard deviation represent three biological replicates.

For CRISPRa, we added a third plasmid (pBR322 ori) constitutively expressing (promoter J23119) MCP-SoxS (carrying mutations R93A+S101A).^22^ We started with a two-node circuit (Figure 1A), with the first node containing an arabinose-inducible scRNA (version b2)^23^ guiding dCas9 and MCP-SoxS to bind and subsequently activate the second node containing a weak promoter (J23117) upstream of a green fluorescent protein (GFP) reporter. As CRISPRa is very sensitive to the distance between the target site and the transcriptional start site (TSS), we used a previously employed sequence ranging from the target site to the TSS (J306 and J3 region).^22^ We confirmed that the previously reported −81 bp distance upstream of the TSS leads to high activation (Figure S1).

Once we had successfully integrated CRISPRa into our framework, we created an orthogonal library of scRNAs (Figure 1A). We added the target-specific sequences of 6 previously characterized orthogonal sgRNAs^27,30^ to the scRNA scaffold and replaced the sequence 81 nt upstream of the TSS with the corresponding binding sites. Four (numbers 1, 4, 5, and 6) out of the six tested constructs resulted in at least 2-fold activation, namely, 2.1–6.1-fold, compared to the noninduced construct and 2.5–23.7-fold activation compared to the off-target control (Figure S2). Together with the original scRNA (number 0), we tested their orthogonality (Figure 1A). This analysis confirmed that we observe activation only when the scRNA and binding site pairs match. We noticed that the noninduced controls of matching pairs resulted in higher green fluorescence levels than nonmatching pairs of scRNA and the binding site (Figure S2). We attribute this to the previously reported leakiness of our pBAD promoter.^27^

### Combining CRISPRa and CRISPRi

Next, we combined CRISPRa and CRISPRi in the same circuit. We added a third node to our activation circuit, which in the presence of AHL produces a sgRNA complementary to a binding site placed downstream of the promoter repressing the expression of GFP in the second node (Figure 1B). We expected that GFP expression increases in the presence of arabinose and decreases in the presence of AHL. In the presence of both inducers, the expression depends on the relative strength of the two opposing inputs, but as the binding site for the CRISPRi complex is downstream of the promoter, we expected the repression to be dominant. However, the circuit showed a very low level of activation in the presence of arabinose only. In our off-target control (orthogonal binding site 2 instead of binding site 1) for inhibition, we observed the expected activation with scRNAs induction, which might indicate that leaky expression of the sgRNA is enough to repress the activation in the full circuit. Moreover, in the off-target control, we noticed a strong repression upon induction of the sgRNA, even though the sgRNA should not repress. These results led us to hypothesize that the sgRNA competes with the scRNA for dCas9, with an advantage for the CRISPRi complex.

### Model Suggests That Differential Affinities of scRNA and sgRNA are Problematic

**Figure 2:**
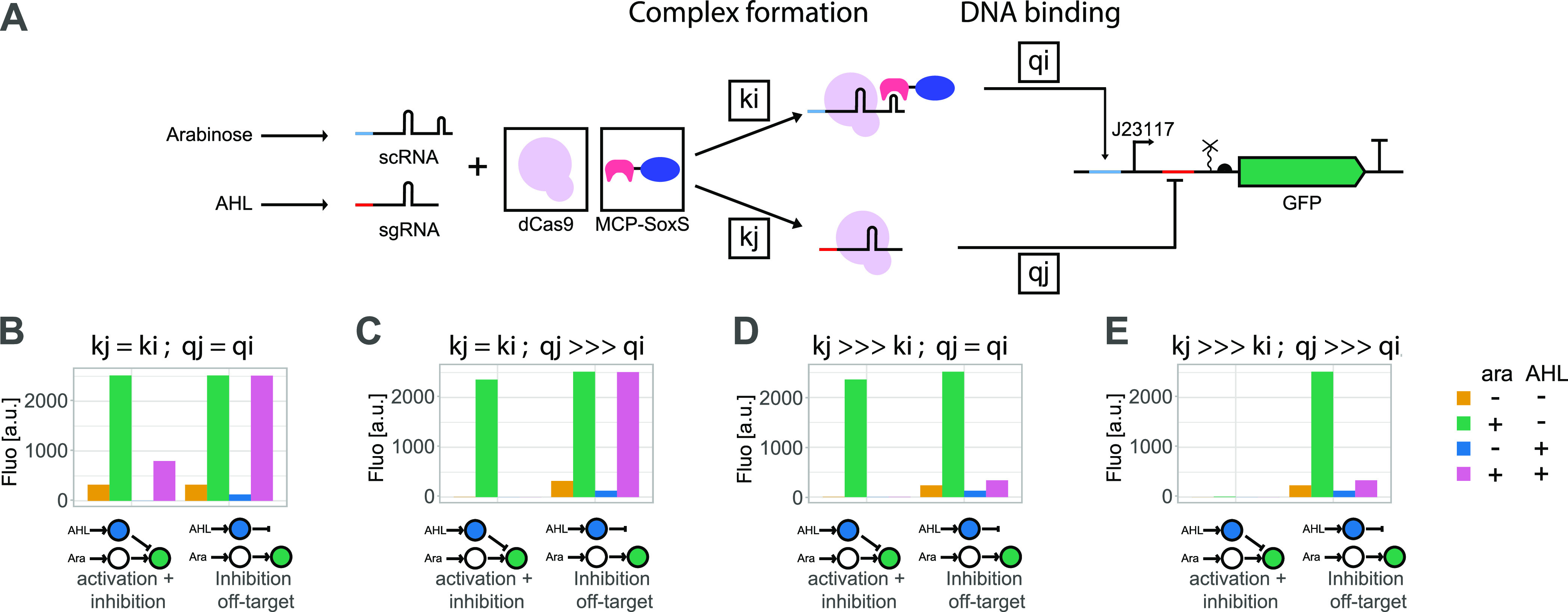
Model suggests that differential affinities of scRNA and sgRNA are a problem. (A) Schematic representation of the quasi-steady-state approximations model. The different elements changed in the analysis are indicated with black boxes: quantity of dCas9 and MCP-SoxS, values of complex formation parameters (*k_i_*, *k_j_*), and values of complex binding to DNA (*q_i_*, *q_j_*). See the Materials and Methods section for detailed equations. (B–E) Qualitative model of GFP intensity with or without arabinose and AHL induction (0,10) and different values of *k_j_* and *q_j_* (1 and 100,000), *k_i_*, and *q_i_* are fixed at 1. For the off-target controls, *q_j_* is set to 0. Situation E resembles most our experimental data. Situations B and C show the desirable behavior.

To test our hypothesis, we adapted a qualitative mathematical model^31^ describing the transcription of scRNA and sgRNAs, the formation of the CRISPRa and CRISPRi complexes, their binding to DNA, and subsequent transcriptional activation or repression, respectively (Figure 2A, see the Materials and Methods section). Then, we varied the key parameters of CRISPRa/i complex formation (*k*_*i*_, *k*_*j*_) and binding of the complexes to DNA (*q*_*i*_,*q*_*j*_) (Figure 2B–E). We found that we could reproduce our experimental finding of Figure 1 when CRISPRi parameters *k*_*j*_ and *q*_*j*_ are several orders of magnitude bigger than their CRISPRa counterparts (*k*_*i*_ and *q*_*i*_) and when we have some leaky expression of sg/scRNAs (Figure 2E). This suggests that the scRNA binds weaker to dCas9 than the sgRNA and that the CRISPRa complex binds weaker to DNA than the CRISPRi complex. Thus, the model supported the hypothesis that scRNA and sgRNA compete for the pool of available dCas9.

Thereby, the model put forward a potential solution to achieve the expected behavior: to ensure the complex formation rates are similar for CRISPRa and CRISPRi (*k*_*i*,*j*_) (Figure 2B,C). The DNA binding affinities (*q*_*i*,*j*_) are less important because dCas9-sgRNA and dCas9-scRNA-MCP-SoxS complexes do not bind to the same binding sites. However, when the complex formation rates are unequal (*k*_*j*_ > > > *k*_*i*_), a very strong binding of the inhibiting CRISPRi complex *q*_*j*_ compared to the activating CRISPRa complex *q*_*i*_ leads to an absence of activation in the on-target circuit (Figure 2E), while similar DNA binding rates *q*_*j*_ = *q*_*i*_ allow for proper activation in the on-target circuit but still show an incorrect off-target behavior (Figure 2D).

We also investigated the influence of dCas9 and MCP-SoxS quantities in our model (Figure S3). Increasing the amount of MCP-SoxS helps to increase the activation level but comes at the price of increased leaky activation in the absence of the inducer (arabinose). Increasing the amount of dCas9 allows the correct behavior of the off-target control but not of the on-target circuit. We hypothesize that the dCas9 increase does not rescue the behavior because of the genetic configuration and the use of slightly leaky promoters. The binding site for inhibition is downstream of the activation binding site. Thus, if both CRISPRa and CRISPRi complexes are bound, then transcription is repressed. The leaky expression of sgRNA and a high concentration of dCas9 are sufficient to inhibit transcription even in the absence of AHL inducer. Therefore, increasing the total amount of dCas9 is not predicted to recover the correct behavior of our circuits. Anyway, high expression levels of dCas9 are known to be toxic to *Escherichia coli* cells,^32^ and MCP-SoxS expression was already maximized with a strong promoter on a high-copy plasmid. Therefore, equalizing the complex formation rates promised to be the most promising approach.

### Using scRNA for CRISPRi and CRISPRa Restores the Function

We thus set out to test the model predictions and attempted to make the complex formation rates similar for CRISPRa and CRISPRi. We first tested whether truncating the sgRNA by 4 bp (sgRNAt4) would lead to the desired behavior (Figure S4A). Truncated sgRNAs-dCas9 complexes display weaker repression than their full-length counterparts,^13^ but the DNA binding of the CRISPR complex is similar as with a full-length sgRNA.^33,34^ We observed the correct behavior for the off-target control but still almost no activation when combined with the on-site repression (Figure S4A). This behavior can be reproduced in our model when sgRNAt4 binds weaker to dCas9 than sgRNA but still stronger than scRNA while DNA binding affinities of sgRNAt4 and sgRNA are similar (Figure S4B).

**Figure 3:**
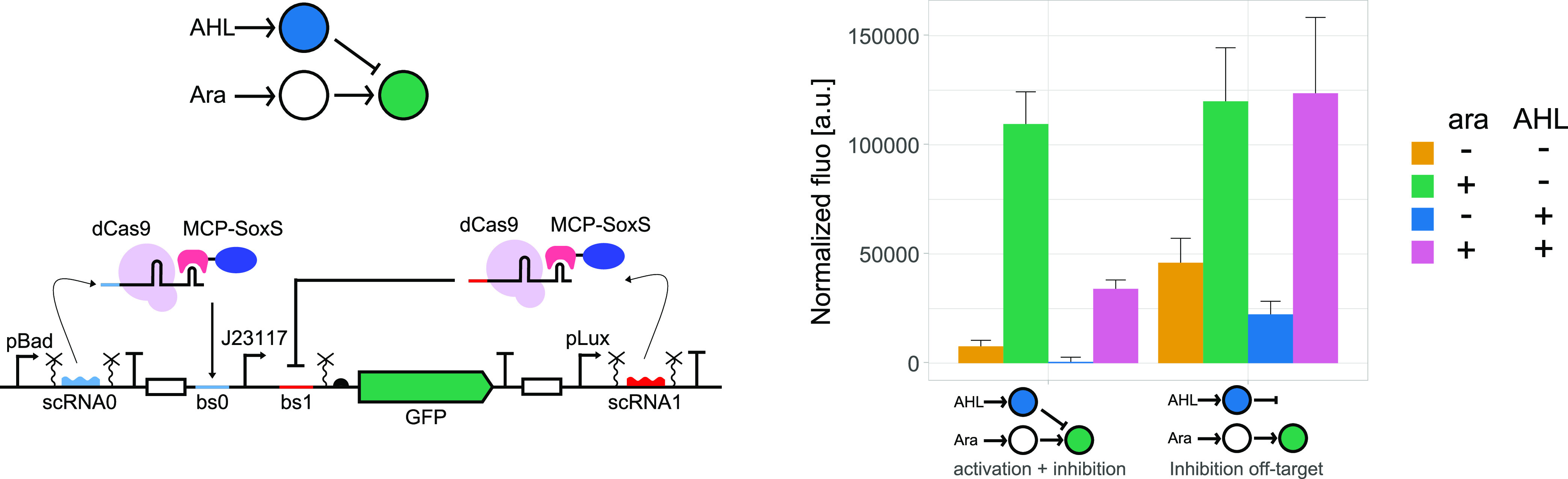
Using scaffold RNA in both CRISPRa and CRISPRi restores predictable circuit behavior. Left: details of the circuit design and schematic representation of the circuit. Right: bar plots represent the GFP fluorescence in the absence or presence of arabinose (0 and 0.2%) and AHL (0 and 0.1 μM). The data shows the correct behavior for both on- and off-target cases: we observe a good level of activation with arabinose induction, and AHL induction of the inhibitory interaction only leads to GFP repression in the on-target case but not for the off-target control. Binding site number 2 was used for off-target inhibition. Mean and s.d. represent three biological replicates.

Next, we used scRNA instead of sgRNA also for the inhibition complex. As observed for other CRISPRa systems,^19,20^ if we directed dCas9-scRNA-MCP-SoxS downstream of a promoter, we observed inhibition rather than activation (Figure S5). We thus rebuilt the circuits in Figure 1, but this time, with scRNAs for both CRISPRa and CRISPRi (Figure 3). Now, we observed a good level of activation in the presence of arabinose in our on-target circuit and no inhibition with AHL induction in the off-target control. These data demonstrate that, in agreement with our model, ensuring similar complex formation rates allows for the correct functioning of combined CRISPRa and CRISPRi circuits. Therefore, using the same scRNAs for both inhibition and activation is a straightforward way to obtain the expected circuit function.

### Cascade Circuits

**Figure 4:**
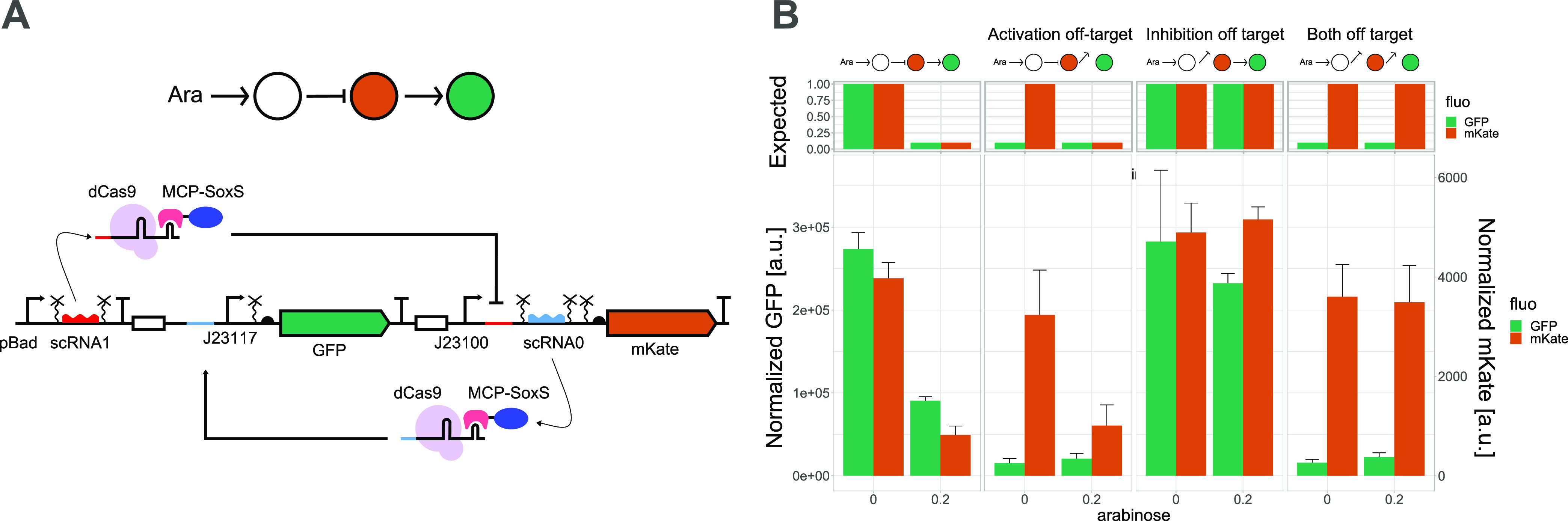
Combination of CRISPRi and CRISPRa in a cascade circuit. (A) Details of the circuit design and schematic representation of the circuit. (B) Bar plot representing the GFP and mKate fluorescences of different circuits with different off-target controls. The red and green fluorescence intensities follow the expected behavior, as illustrated at the top. Mean and s.d. represent three biological replicates.

Encouraged by the predictable behavior when scRNA was used for both CRISPRa and CRISPRi complexes, we proceeded to combine CRISPRa and CRISPRi in a cascade circuit. Here, the first node is induced by arabinose, and it represses the second node (containing a mKate reporter) that activates the third node encoding a GFP reporter (Figure 4A). This circuit also behaved as expected: we observed expression of GFP and mKate in the absence of arabinose, and their level decreased upon addition of arabinose (Figure 4B). In addition, we built three off-target controls. All measurements of the controls agreed with our expectations, while using sgRNA for inhibition led again to an incorrect behavior of the off-target controls (Figure S6). We thus demonstrated that CRISPRa and CRISPRi can be successfully combined, but special attention has to be paid to different affinities in RNA-dCas9 complex formation and DNA binding.

## Discussion

In this work, we successfully implemented the SoxS-based CRISPRa system first described by Fontana et al.^22^ into our framework.^27,35^ When we combined CRISPRa with CRISPRi, we observed a strong affinity competition for dCas9, leading to weak activation in on-target circuits and an incorrect circuit function of the off-target controls. This can lead to an undesirable coupling among circuit branches that theoretically should act orthogonal. Guided by mathematical modeling, we managed to avoid this problem and obtained the circuits’ correct function by using the same RNA (i.e., scRNA) for CRISPRa and CRISPRi resulting in the same complex affinities for activation and inhibition.

Competition for transcriptional and translational resources is a well-known issue in engineering synthetic circuits.^9,36^ Moreover, it has also been shown that expressing simultaneously multiple sgRNAs that compete for the same limited pool of dCas9 can lead to unwanted outcomes.^31,37^ Here, we describe yet another problem related to dCas9 resource competition when combining CRISPRi (using sgRNA) and CRISPRa (using scRNA) in one bacterial cell. The different guide RNAs have different affinities for dCas9, and thus, competition hampers the correct network function. Previous work by Tickman and colleagues combined CRISPRi and CRISPRa in different circuits such as cascades and incoherent feed-forward circuits in cell-free extract and in *E. coli*.^24^ However, they did not report a resource competition between the two systems. It might be that they had conditions with very tight sgRNA production and high dCas9 concentrations where competition was minimized.

While building synthetic circuits with CRISPRi and CRISPRa is rather new in prokaryotes, the combination of CRISPRa and CRISPRi has been used to control host genes in yeast and mammalian cells.^38−43^ In these systems, the competition between CRISPRa and CRISPRi paths was not observed, as they either used orthogonal Cas proteins^38−40^ or scRNAs for CRISPRa and CRISPRi as both functions require regulator domains.^41−43^

Here, we present a simple solution to the encountered problem in *E. coli* by using the same complex for both CRISPRa and CRISPRi. Depending on whether we place its binding site upstream or downstream of the promoter, we observe activation or repression, respectively. This is akin to how some protein transcription factors, such as LuxR, have been used as activators and repressors.^44^ An alternative approach could be to design a new guide RNA with a similar dCas9 affinity as the scRNA, but without binding MCP-SoxS. This would reduce the required expression level of MCP-SoxS. To further reduce the metabolic burden on the cells, one could express dCas9 and the MCP-SoxS from the genome. This would reduce the number of plasmids used and free resources to maintain them. However, this would require readjusting the strengths of promoters and/or ribosomal binding sites to get a sufficiently high level of expression.

Our model suggests that simply increasing the dCas9 pool cannot restore the correct function in our circuits (Figure S3). This is due to the leaky production of sgRNA and the dominant effect of repression over activation caused by the circuit architecture. While increasing the concentration of dCas9 may help in other cases of dCas9 competition, overproduction of dCas9 can lead to toxicity, reduced growth rates, and morphological defects.^32,37^ Reported solutions to address this problem include the use of a nontoxic variant of dCas9^37^ and regulated production of dCas9 adapting to the current circuit load.^45^ For future work, it would be interesting to test some of these approaches in CRISPRa/i circuits. Moreover, to further increase the complexity of functions that can be programmed, it would be exciting to combine these circuits with guide RNAs that can be controlled by small molecules^46^ or RNAs.^47^ We hope our work paves the way for building more complex bacterial CRISPRa/i circuits and their applications for studying the function of native genes, cellular reprogramming, and metabolic engineering.^40,48−50^

## Materials and Methods

### Construction of Plasmids

Circuits were built as previously described.^28^ The different parts contain prefix (CAGCCTGCGGTCCGG) and suffix (TCGCTGGGACGCCCG) sequences,^51^ which can be PCR-amplified (Phanta Max Super-Fidelity DNA Polymerase, Vazyme) with a set of primers (ordered from Microsynth or Sigma-Aldrich) to add a unique variable linker. Backbones were linearized with PCR or with restriction enzymes (NEB, 1 h at 37 °C). PCR-amplified or digested products were purified (Monarch PCR & DNA Cleanup Kit, NEB). Then, the parts were assembled with Gibson assembly (NEBuilder HiFi DNA Assembly Master Mix from NEB, 1 h, at 50 °C) with the linkers providing sequence overlaps. Finally, 1 μL of the assembly mixes were transformed into competent cells (NEB5α cell) by electroporation and plated on LB agar plate containing appropriate antibiotics (50 μg/L kanamycin, 100 μg/L ampicillin, or 50 μg/L spectomycin). The obtained plasmids were sequenced (Microsynth) to confirm that they contained the correct constructs. A list of all plasmids and complete plasmid sequences is provided as Supporting Information.

### Fluorescence Measurements

Plasmids were cotransformed into Mk01 *E. coli* cells.^52^ Single colonies were incubated in 200 μL of EZ medium with 0.4% glycerol as carbon source (Teknova) with appropriate antibiotics (25 μg/L kanamycin, 50 μg/L ampicillin, and 25 μg/L spectomycin) at 37 °C, 200 rpm, for 4–5 h. Then, cells were diluted to 0.05 OD_600_ in a 96-well CytoOne plate (Starlab) with or without inducers, as indicated in the figures. Plates were incubated at 37 °C with double-orbital shaking (Synergy H1 microplate reader, Biotek, with Gen5 3.04 software). Fluorescence was determined after 16 h with 479 nm excitation and 520 nm emission for GFP and 588 nm excitation and 633 nm emission for mKate2. We subtracted a blank (medium only) from all fluorescence and absorbance values, and the resulting cellular fluorescence values were divided by the absorbance at 600 nm of the same sample to correct for differences in bacterial concentration. After that, the bacterial autofluorescence of a control with nonfluorescent cells (3 replicates) was subtracted. In particular, we used the following formula: (GFP – blankGFP)/(OD – blankOD) - mean((autofluoGFP – blankGFP)/(autofluoOD – blankOD)). Subsequent data were analyzed and visualized with R.

### Modeling

The model is based on mass action law kinetics and quasi-steady-state approximations (QSSA) accounting for various molecular steps and constraints such as copy number of plasmids and steady-state protein levels. Mass action law kinetics states that rates of reactions are dependent on the concentrations of the reactants.^53^ QSSA laws state that the concentration of enzyme–substrate complexes remains almost constant, and the rate of change of said enzyme–substrate complexes is extremely small.^54^ This approximation enables us to not go into the details of forward and backward reactions in complex formations and figure out their steady-state concentrations by using mass action law kinetics. Moreover, we assume that the binding of sgRNA/scRNA in inhibiting and activating edges is independent of each other. The model is explained below, where the set of equations with subscript *i* refers to activation due to arabinose, and *j* refers to inhibition due to AHL. The parameters used are explained in Table 1. The code is available at https://github.com/SchaerliLab/CRISPRa-i-circuits

**Table 1:** Parameters’ Description and Values

parameter	description	value	unit
δ_RNA_	degradation constant for first-order degradation of guide RNA	10.8	hr^–1^
γ_*i*,*j*_	transcription rates of guide RNA/scRNA (competing guide RNAs)	530.1	nM hr^–1^
*K*_*j*_	transcription rate due to leaky promoter	5	hr^–1^
*K*_*i*_	transcription rate from dCas9 bound promoter (activation)	100	hr^–1^
δ_GFP_	degradation constant for first-order degradation of GFP RNA	1.176	hr^–1^
*D*_*i*,*j*_	concentration of free target DNA binding sites for scRNA (*i*) or sgRNA (*j*)	variable	nM
*D*_*i*_,*j*_total_	total concentration of target DNA binding sites for scRNA (*i*) or sgRNA (*j*)	30	nM
*d*_total_	total quantity of dCas9	100 or 10,000	
*m*_total_	total quantity of MCP-SoxS	100	
*k*_*i*_	scRNA (*x*_*i*_), dCas9, and MCP-SoxS complex formation affinity	1	
*k*_*j*_	sgRNA (*x*_*j*_) and dCas9 complex formation affinity	variable (1–100,000)	
*q*_*i*_	DNA binding affinity of the CRISPRa complex	1	
*q*_*j*_	DNA binding affinity of CRISPRi complex	1 or 100,000	
*x*_*i*_	concentration of scRNA	variable	
*x*_*j*_	concentration of sgRNA	variable	

Rate of change of scRNA and sgRNA from arabinose and AHL induction, respectively
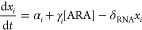
1
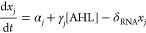
2Here, we have scRNA and sgRNA concentrations changing over time due to leaky basal production denoted by α, a first-order production in the presence of arabinose and AHL, respectively (denoted by production rate γ), and a first-order degradation of RNA strands (denoted by δ_RNA_).

Formation of CRISPRa and CRISPRi complexes (lacking DNA binding sites)
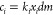
3
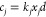
4For the activation complex, free dCas9 and MCP-SoxS bind to the scRNA in a ternary complex, the steady-state concentration of which is given by eq 3. The inhibitory complex utilizing sgRNA involves dCas9 binding to sgRNA eq 4. However, for the case where inhibition utilizes scRNA, the complex concentration is similar to eq 3 as MCP-SoxS is also involved in the complex formation.

Formation of CRISPRa and CRISPRi complexes with DNA binding sites
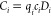
5
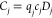
6Once the CRISPRi and CRISPRa complexes have been formed, the search for free complementary sequences (denoted by D) occurs, and the final transcriptional complexes are obtained from mass action law kinetics.

Constraints on DNA binding sites
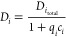
7
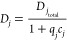
8At any given point, the average total number of binding sites in a cell is the plasmid copy number (denoted by *D*_*i*_total__ and *D*_*j*_total__). Some of these sites are free of any transcriptional complex (*D*_*i*_ and *D*_*j*_), while others are sites of transcriptional repression or activation (*C*_*i*_ and *C*_*j*_). After transcriptional complex concentrations were inserted from eqs 5 and 6, this constraint is depicted in eqs 7 and 8.

Rate of production of GFP from activation due to CRISPRa, leaky expression, and first-order RNA degradation
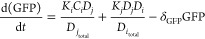
9

In our experimental system, we would expect the maximum production of GFP when CRISPRa is causing proper activation of the promoter (i.e., complex *C*_*i*_), and there is no inhibition due to CRISPRi (complex *D*_*j*_). In our model, we implement this using principles of conditional probability and obtain the net number of such sites as , which, multiplied by the production rate of GFP RNA gives us the first term of eq 9. We also expect leaky production of GFP RNA when both CRISPRa and CRISPRi are inactive. The total number of such sites available are , which is multiplied by the rate of basal expression (*K*_*i*_) to capture leaky expression in our model. We assume that any presence of the CRISPRi complex will lead to a complete block of transcription. It is important to note that the values of *D*_*i*_total__ and *D*_*j*_total__ are the same in our system, as both our sites are present on the same plasmid.

Constraint equations for dCas9 and SoxS proteins:
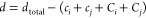
10
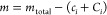
11

In the equations above, *x*_*i*,*j*_ are the concentration of scRNA(i) and sgRNA(j), *c*_*i*_ is the concentration of scRNA, dCas9, and MCP-SoxS complex, *c*_*j*_ is the concentration of the sgRNA and dCas9 complex, *C*_*i*,*j*_ is the activation or inhibition complex bound to DNA, *D*_*i*,*j*_ is the amount of free DNA binding sites, *d* is the concentration of free dCas9, and *m* is the concentration of free MCP-SoxS. The descriptions and values of the different parameters are summarized in Table 1. Due to the lack of studies on the biochemical properties of CRISPRi and CRISPRa, arbitrary values were chosen for all parameters not available in the literature. The model was updated with a simplistic Euclidean update and brent equation solver in python3.

## Supporting Information Available

Activation sensitivity to the distance to the transcriptional start site, induction of CRISPRa at different arabinose concentrations, modeling the effect of dCas9 and SoxS concentrations, CRISPRa/i circuit with different guide RNAs for CRISPRi, inhibition with sgRNA or scRNA, cascade circuit with sgRNA and scRNA (PDF)
List of plasmids and plasmid sequences (ZIP)
