# Supplementary material for: Synthetic Gene Circuits Combining CRISPR Interference and CRISPR Activation in E. coli: Importance of Equal Guide RNA Binding Affinities to Avoid Context-Dependent Effects
Source: ACS Synth Biol. 2023 Oct 9;12(10):3064–71. doi: 10.1021/acssynbio.3c00375 (PMC10594877; doi:10.1021/acssynbio.3c00375)
Supplement: Supplementary file 1 — sb3c00375_si_001.pdf [file sb3c00375_si_001.pdf]

Supporting Information

Synthetic gene circuits combining CRISPR interference and CRISPR activation in *E. coli*: importance of equal guide RNA binding affinities to avoid context-dependent effects

Içvara Barbier[1], Hadiastri Kusumawardhani[1,+], Lakshya Chauhan[1,2,+], Pradyumna Vinod Harlapur[2], Mohit Kumar Jolly[2], and Yolanda Schaerli[1,*]

[1]Department of Fundamental Microbiology, University of Lausanne, Switzerland
[2]Centre for BioSystems Science and Engineering, Indian Institute of Science, India
[+]These authors contributed equally to the project
[*]Corresponding author: yolanda.schaerli@unil.ch

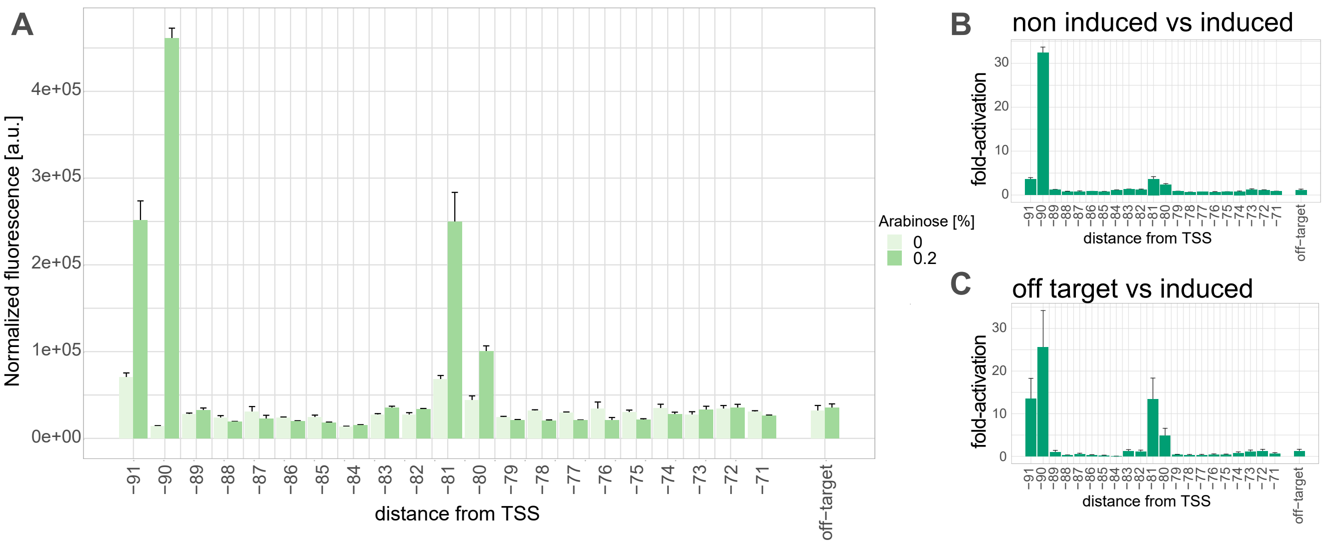

Figure S1: Activation is sensitive to the distance to the transcriptional start site (TSS)

A. *Normalized green fluorescence at indicated arabinose concentrations. Different binding site position upstream of the TSS were tested as indicated in the x axis.* ***B.*** *Fold-activation at 0.2% arabinose compared to fluorescence level at 0% arabinose.* ***C.*** *Fold-activation at 0.2% arabinose compared to off-target construct (scRNA 1 - binding site 4) at 0% arabinose. Mean and s.d. represent three biological replicates.*

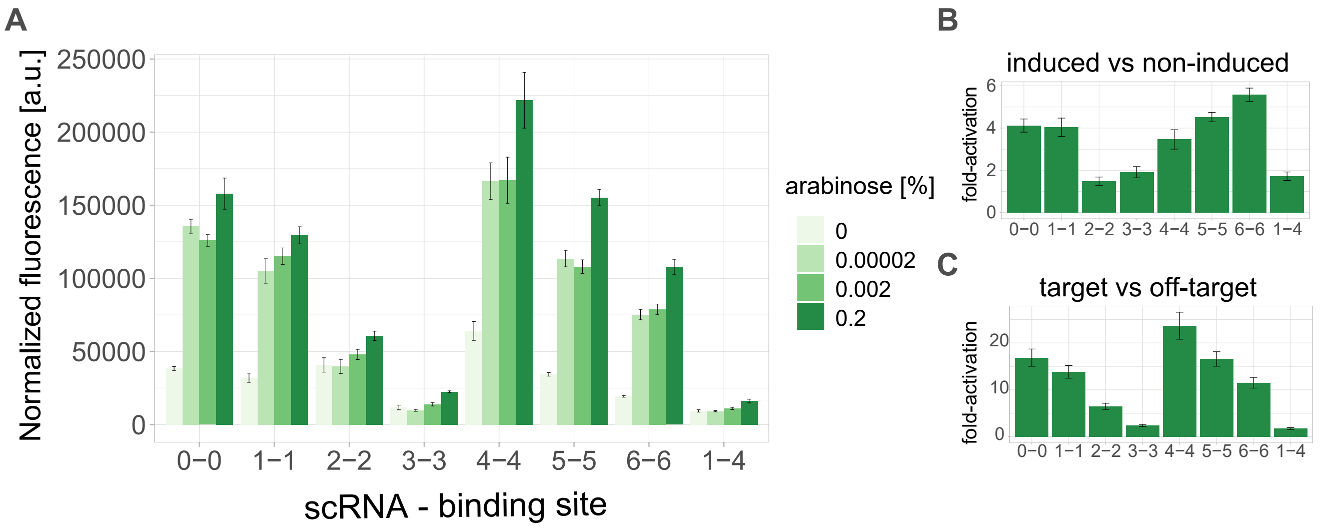

Figure S2: Induction of CRISPRa at different arabinose concentrations
A. *Normalized green fluorescence at indicated arabinose concentrations. Different combinations of scRNAs and binding sites were tested as indicated in the x axis.* ***B.*** *Fold-activation at 0.2% arabinose compared to fluorescence level at 0% arabinose.* ***C.*** *Fold-activation at 0.2% arabinose compared to off-target construct (scRNA 1 - binding site 4) at 0% arabinose. Mean and s.d. of three biological replicates.*

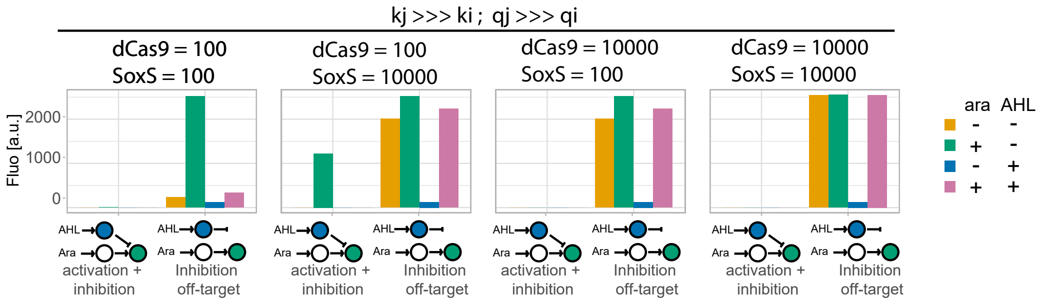

Figure S3: Increasing dCas9 and SoxS quantities does not lead to correct function.
Qualitative model of GFP intensity with or without arabinose and AHL induction (0,10) and different values of MCP-SoxS and dCas9. kj and qj are fixed at 100000, ki and qi are fixed at 1. For the off-target controls, qj is set to 0.

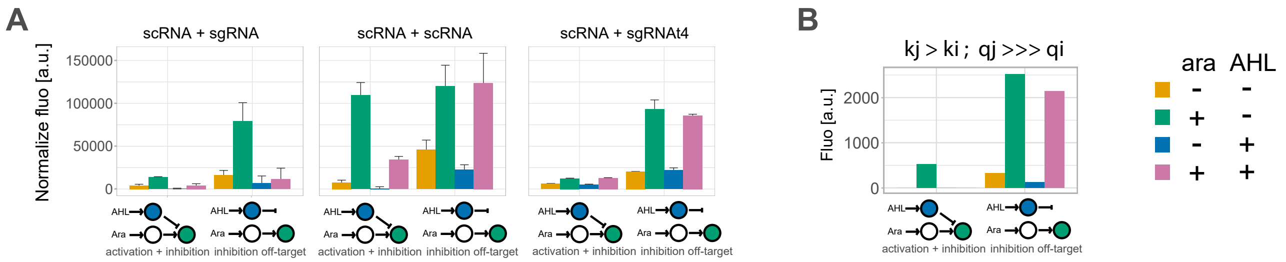

Figure S4: CRISPRa/i induction with different guide RNAs for CRISPRi

A. *CRISPRa with scRNA and CRISPRi with sgRNA, scRNA or sgRNAt4 in on-target circuits or off-target controls. Bar plots at 0 or maximum concentration of AHL (0.1 μM) and arabinose (0.2%). Mean and s.d. represent three biological replicates.**B.** Modeling as in figure 2 but with qj = 100000 and kj = 1000*

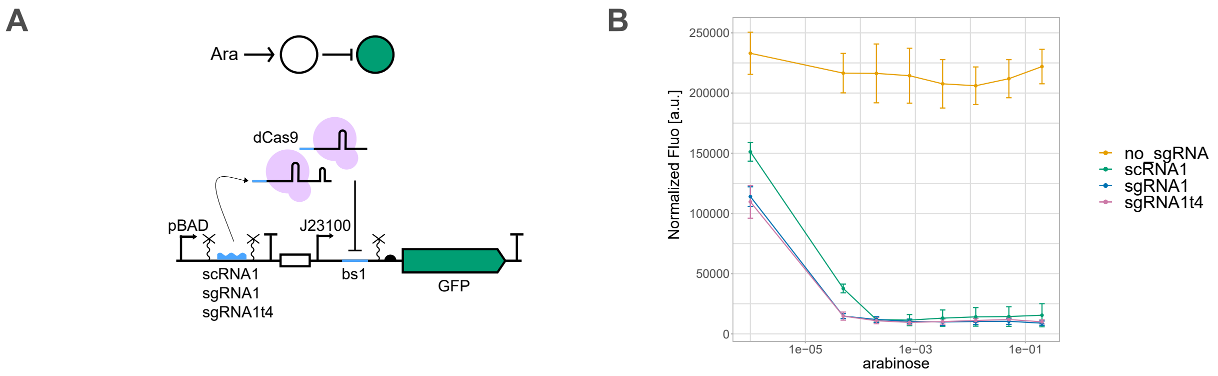

Figure S5: Inhibition with sgRNA or scRNA

A. *Details of the circuit design and schematic representation of the circuit.* ***B.*** *GFP fluorescence in presence of different concentrations of arabinose. Mean and s.d. represent three biological replicates.*

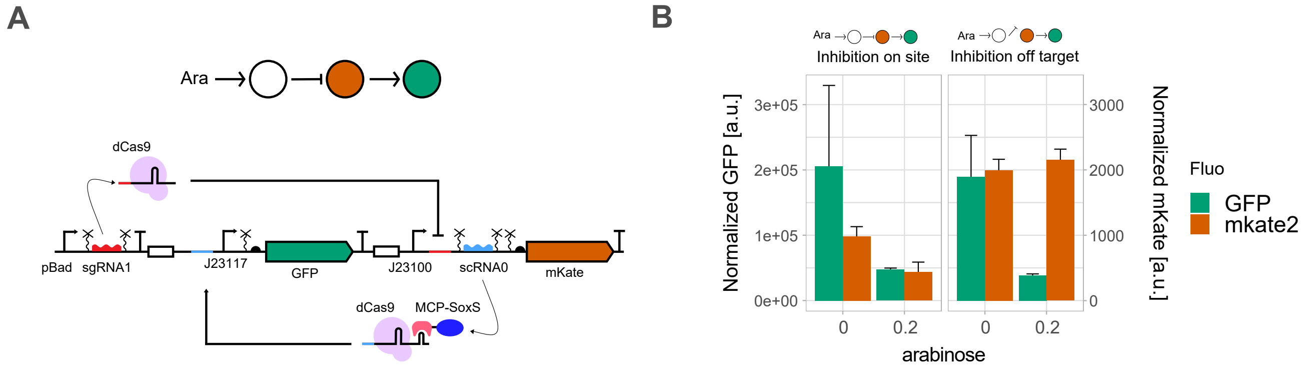

Figure S6: Cascade circuit with sgRNA and scRNA leads to unpredicted behavior of the off-target control ***A.*** *Details of the circuit design and schematic representation of the circuit. It is the same as in Figure 4, but instead of using scRNA1, we used here sgRNA1* ***B.*** *Bar plots represent the GFP and mKate fluorescences of the circuit and of an off-target control in absence (0%) or presence of arabinose (0.2%). Mean and s.d. represent three biological replicates.*
